# Two new species of *Hyaloambispora* and *Paramirandina* (Microthyriaceae, Microthyriales) from freshwater habitats in China

**DOI:** 10.3897/mycokeys.136.199668

**Published:** 2026-07-15

**Authors:** Lei Shao, Yong Liu, Yuxin Shi, Yu Liu, Wensheng Yang, Jian Ma

**Affiliations:** 1 College of Pharmacy, Guiyang KangYang University, Guiyang, Guizhou 550081, China School of Food and Pharmaceutical Engineering, Guizhou Institute of Technology Guiyang China; 2 Guizhou Key Laboratory of Agricultural Microbiology, Guiyang, Guizhou 550000, China College of Pharmacy, Guiyang KangYang University Guiyang China; 3 Guizhou Provincial Engineering Research Centerof Medical Resourceful Health Care Products, Guiyang KangYang University, Guiyang, Guizhou 550081, China Guizhou Key Laboratory of Agricultural Microbiology Guiyang China; 4 Guizhou Industry Polytechnic University, Guiyang, Guizhou 550008, China Guizhou Provincial Engineering Research Centerof Medical Resourceful Health Care Products, Guiyang KangYang University Guiyang China; 5 School of Food and Pharmaceutical Engineering, Guizhou Institute of Technology, Guiyang, Guizhou 550003, China Guizhou Industry Polytechnic University Guiyang China

**Keywords:** Asexual morph, Dothideomycetes, freshwater fungi, phylogeny, taxonomy

## Abstract

During a survey of the taxonomy and diversity of saprobic fungi, fresh samples were collected from submerged decaying wood in freshwater habitats in Guizhou and Hainan Provinces, southern China. Phylogenetic analyses, based on LSU, ITS, SSU, *tef*1-α and *rpb*2 sequence data, together with morphological characteristics, support the introduction of two new species, *Hyaloambispora aquatica***sp. nov**. and *Paramirandina fusiseptata***sp. nov**. Detailed morphological descriptions, illustrations and multi-locus phylogenetic analyses are provided herein to support and substantiate the taxonomic placement of these newly-introduced taxa. Importantly, this study represents the first record of *Hyaloambispora* species from Guizhou Provinces, China, further expanding the known diversity of freshwater fungi in these regions.

## Introduction

Microthyriales was established by Arnaud (1918), based on its distinctive circular thyriothecial ascomata. Microthyriaceae was introduced by Saccardo (1883) with *Microthyrium* as the type genus. Currently, 20 genera were accepted in Microthyriaceae, including *Antidactylaria*, *Arnaudiella*, *Chaetothyriothecium*, *Hamatispora*, *Heliocephala*, *Hyaloambispora*, *Isthmomyces*, *Keqinzhangia*, *Microthyrium*, *Neoanungitea*, *Nothoanungitopsis*, *Nothomicrothyrium*, *Paramicrothyrium*, *Paramirandina*, *Pseudocorniculariella*, *Pseudocoronospora*, *Pseudomicrothyrium*, *Pseudopenidiella*, *Triscelophorus* and *Tumidispora* (Hyde et al. 2024; Xu et al. 2025).

Amongst these genera, *Hyaloambispora* was recently established by Xu et al. (2025), based on its distinctive fusiform, reniform or obpyriform conidia and supported by multigene phylogenetic analyses. To date, *Hyaloambispora* is known exclusively from its asexual morph and no sexual morph has been observed or reported. Currently, the genus comprises two species, namely *Hyaloambispora drungiorum* and *H. reniformis*, both of which were described from freshwater habitats in north-western Yunnan Province, China, highlighting the ecological preference of the genus for aquatic environments (Xu et al. 2025).

*Paramirandina* was established by Liu et al. (2023), with *P. aquatica* designated as the type species. *Paramirandina* is characterised by macronematous, mononematous, unbranched, erect, cylindrical, septate conidiophores that are dark brown, becoming pale brown to subhyaline towards the apex; polyblastic, cylindrical to lageniform, pale brown to subhyaline conidiogenous cells that may elongate percurrently; and solitary or catenate, fusiform, cymbiform or narrowly lunate, hyaline, septate conidia (Liu et al. 2023). Currently, the genus comprises three species, *Paramirandina aquatica*, *P. cymbiformis* and *P. guttulata*, all of which have been reported from freshwater habitats in China (Liu et al. 2023; Shen et al. 2024).

During investigations of freshwater fungi in southern China, four hyphomycetous isolates, representing two distinct taxa, were collected from submerged decaying wood in Guizhou and Hainan Provinces. To determine their taxonomic placement, detailed morphological observations and phylogenetic analyses, based on combined LSU, ITS, SSU, *tef*1-α and *rpb*2 sequence data, were conducted. Based on morphological characteristics and multigene phylogenetic evidence, two novel species, *Hyaloambispora aquatica* and *Paramirandina fusiseptata*, are introduced herein.

## Materials and methods

### Sample collection, examination and isolation

Decaying wood samples were collected from freshwater habitats in Guizhou and Hainan Provinces, southern China, between 2 September 2021 and 22 May 2025. Fresh samples were transported to the laboratory in plastic bags with the collection details, including localities, habitats and dates (Rathnayaka et al. 2025). Samples were cultured in plastic boxes, lined with moistened tissue at room temperature for 1–2 weeks. The microscopic features were examined and photographed using a stereomicroscope (SMZ-168, Nikon, Japan) and an ECLIPSE Ni compound microscope (Nikon, Tokyo, Japan) with a Canon 90D digital camera. Measurements were made using Tarosoft (R) Image Frame Work software. Photo-plates were made using Adobe Photoshop 2019 programme (Adobe Systems, USA) and Adobe Illustrator version 51.1052.0.0 (Adobe Inc., San Jose, California, USA).

Single spore isolations were done on PDA (potato dextrose agar) plates following the methods described by Senanayake et al. (2020) and the germinated asexual conidia were aseptically transferred to fresh PDA plates. Morphological characteristics of fungal mycelia on PDA, including colony colour, hyphal shape and growth dimensions, were recorded. Dried fungal specimens were deposited in the Herbarium of Guizhou Academy of Agriculture Sciences (Herb. GZAAS), Guiyang, China. Pure cultures were preserved in the Guizhou Culture Collection, China (GZCC), Guiyang, China. The MycoBank numbers were obtained as described in https://www.mycobank.org/.

### DNA extraction, PCR amplification and sequencing

Fresh fungal mycelia were scraped from colonies grown on PDA plates and transferred to a 1.5 ml microcentrifuge tube using a sterilised lancet for genomic DNA extraction. Genomic DNA was extracted using the Biospin Fungus Genomic DNA Extraction Kit (BioFlux, China). The primer pairs LR0R/LR5 (Vilgalys and Hester 1990), ITS5/ITS4 (White et al. 1990), NS1/NS4 (White et al. 1990), EF1-983F/EF1-2218R (Rehner and Buckley 2005) and fRPB2-5F/fRPB2-7cR (Liu et al. 1999) were used to amplify the LSU, ITS, SSU, *tef*1-α and *rpb*2 regions, respectively. DNA preparation was conducted in a 25 μl mixture, which included 1 μl of DNA, 1 μl of each forward and reverse primer and 22 μl of 1.1× T3 Supper PCR Mix (Qingke Biotech, Chongqing, China). The PCR conditions for LSU, ITS, SSU and *tef*1-α amplification were as follows: initial denaturing step of 95 °C for 5 min, followed by 35 cycles of denaturation at 94 °C for 30 s, annealing at 52 °C for 45 s, elongation at 72 °C for 1 min and final extension at 72 °C for 10 min. The PCR conditions for *rpb*2 amplification was as follows: initial denaturing step of 95 °C for 5 min, followed by 40 cycles of denaturation at 95 °C for 1 min, annealing at 55 °C for 2 min, elongation at 72 °C for 90 s and final extension at 72 °C for 10 min. The PCR products were purified and sequenced by Sangon Biotech (Shanghai, China) Co., Ltd.

### Phylogenetic analyses

The newly obtained sequences were quality-checked and assembled using BioEdit v.7.0.5.3 (Hall 1999) and SeqMan v.7.0.0 (DNASTAR, Madison, WI, USA; Swindell and Plasterer 1997), respectively. The sequences used in this study were retrieved from GenBank (Table 1; https://www.ncbi.nlm.nih.gov/). Sequence matrices for each gene were aligned using MAFFT v.7.473 (https://mafft.cbrc.jp/alignment/server/; Katoh et al. 2019). Each gene dataset was trimmed using trimAl v.1.2rev59 software (Capella-Gutiérrez et al. 2009). A concatenated sequence dataset was generated using SequenceMatrix-Windows-1.7.8 software (Vaidya et al. 2011).

**Table 1.** Taxa used in this study and their GenBank accession numbers.

**Taxon**	**Strain**	**GenBank accessions**				
		**LSU**	**ITS**	**SSU**	***tef*1-α**	***rpb*2**
*Antidactylaria ampulliforma*	CBS 223.59	MH869386	MH857845	N/A	N/A	N/A
***Antidactylaria minifimbriata***	**CGMCC 3.18825**	**MK577808**	**MK569506**	**MK577793**	**MK577838**	**MK577823**
***Anungitopsis speciosa***	**CBS 181.95**	**EU035401**	**EU035401**	**N/A**	**N/A**	**N/A**
***Chaetothyriothecium elegans***	**CPC 21375**	**KF268420**	**N/A**	**N/A**	**N/A**	**N/A**
***Condylospora vietnamensis***	**NBRC 107639**	**LC146725**	**LC146723**	**N/A**	**N/A**	**N/A**
***Hamatispora phuquocensis***	**VICCF 1219**	**LC064073**	**LC064074**	**N/A**	**N/A**	**N/A**
*Heliocephala elegans*	MUCL 39003	HQ333478	HQ333478	**N/A**	**N/A**	**N/A**
*Heliocephala gracilis*	MUCL 41200	HQ333479	HQ333479	**N/A**	**N/A**	**N/A**
***Heliocephala natarajanii***	**MUCL 43745**	**HQ333480**	**HQ333480**	**N/A**	**N/A**	**N/A**
***Heliocephala zimbabweensis***	**MUCL 40019**	**HQ333481**	**HQ333481**	**N/A**	**N/A**	**N/A**
***Hyaloambispora aquatica***	**GZCC 27-27604**	**PZ322277**	**N/A**	**N/A**	**PZ559356**	**N/A**
*Hyaloambispora aquatica*	GZCC 27-27605	PZ322278	N/A	N/A	PZ559357	N/A
***Hyaloambispora drungiorum***	**HKAS 136188**	**PQ455251**	**OP626339**	**PQ218213**	**N/A**	**N/A**
***Hyaloambispora reniformis***	**HKAS 136189**	**PQ455250**	**OP626350**	**PQ218212**	**N/A**	**N/A**
*Isthmomyces dissimilis*	CGMCC 3.18826	MK577811	MF740794	MK577796	MK607171	MK577826
*Isthmomyces lanceatus*	CBS 622.66	MH870563	MH858897	MK577798	MK607173	MK577828
*Isthmomyces macrosporus*	CGMCC 3.18824	MK577812	MF740796	MK577797	MK607172	MK577827
***Isthmomyces oxysporus***	**CGMCC 3.18821**	**MK577810**	**MF740793**	**MK577795**	**MK607170**	**MK577825**
*Keqinzhangia aquatica*	YMF 1-04262	MK577809	MK569507	NG_242399	MK577839	MK577824
***Kirschsteiniothelia acutispora***	**MFLU 21-0127**	**ON980758**	**OP120780**	**ON980754**	**N/A**	**OP009582**
*Kirschsteiniothelia lignicola*	MFLUCC 10-0036	HQ441568	HQ441567	HQ441569	N/A	N/A
***Lichenopeltella pinophylla***	**CBS 143816**	**MG844152**	**N/A**	**N/A**	**N/A**	**N/A**
***Microthyrium buxicola***	**MFLUCC 15-0212**	**KT306551**	**N/A**	**N/A**	**N/A**	**N/A**
*Microthyrium fici-septicae*	MFLUCC 20-0174	MW063252	N/A	N/A	N/A	N/A
*Microthyrium microscopicum*	CBS 115976	GU301846	N/A	N/A	N/A	N/A
***Microthyrium propagulensis***	**IFRD 9037**	**KU948989**	**N/A**	**N/A**	**N/A**	**N/A**
***Natipusilla decorospora***	**AF236-1**	**HM196369**	**N/A**	**HM196376**	**N/A**	**N/A**
***Natipusilla naponense***	**AF217-1**	**HM196371**	**N/A**	**HM196379**	**N/A**	**N/A**
***Neoanungitea eucalypti***	**CBS 143173**	**MG386031**	**MG386031**	**N/A**	**N/A**	**N/A**
***Neoscolecobasidium agapanthi***	**CPC 28778**	**NG_059748**	**NR_152546**	**N/A**	**N/A**	**N/A**
***Nothoanungitopsis urophyllae***	**CBS 146799**	**MW883825**	**MW883433**	**N/A**	**N/A**	**N/A**
***Ochroconis dracaenae***	**CPC 26115**	**KX228334**	**KX228283**	**N/A**	**N/A**	**KX228370**
***Paramirandina aquatica***	**GZCC 19-0408**	**OQ025201**	**OQ025199**	**OQ025204**	**OQ032664**	**OQ032662**
***Paramirandina cymbiformis***	**HKAS 112619**	**OQ025202**	**N/A**	**OQ025205**	**OQ032665**	**OQ032663**
***Paramirandina fusiseptata***	**GZCC 27-27606**	**PZ322279**	**PZ534498**	**N/A**	**PZ559358**	**PZ542406**
*Paramirandina fusiseptata*	GZCC 27-27607	PZ322280	PZ534499	N/A	PZ559359	PZ559355
***Parazalerion indica***	**CBS 125443**	**MH874977**	**MH863483**	**N/A**	**N/A**	**N/A**
***Phaeotrichum benjaminii***	**CBS 541.72**	**MH872266**	**MH860561**	**AY538349**	**DQ677892**	**DQ677946**
*Pleurotheciopsis bramleyi*	CBS 127863	N/A	MH877952	**N/A**	**N/A**	**N/A**
***Pleurotheciopsis tropicalis***	**CBS 100511**	**MH862705**	**MH874311**	**N/A**	**N/A**	**N/A**
***Pseudocorniculariella guizhouensis***	**GZCC 19-0513**	**OQ025203**	**OQ025200**	**N/A**	**OQ032666**	**N/A**
*Pseudocoronospora hainanensis*	YMF 1-04517	MK577807	MK569505	MK577792	MK577837	MK577822
*Pseudomicrothyrium thailandicum*	MFLU 14-0286	MT741680	N/A	NG_081398	N/A	N/A
*Pseudopenidiella gallaica*	CBS 121796	LT984843	LT984842	N/A	N/A	N/A
***Pseudopenidiella piceae***	**CBS 131453**	**JX069852**	**JX069868**	**N/A**	**N/A**	**N/A**
***Pseudosoloacrosporiella cryptomeriae***	**CBS 148441**	**NG_081320**	**NR_175206**	**N/A**	**OK651183**	**N/A**
***Scolecopeltidium menglaense***	**MFLU 19-1009**	**MW003710**	**MW003724**	**N/A**	**N/A**	**N/A**
***Scolecopeltidium wangtianshuiense***	**IFRD 9302**	**NG_067860**	**NR_166263**	**N/A**	**N/A**	**N/A**
*Seynesiella juniperi*	I1201	MW405232	MW405223	N/A	MW619850	MW619847
*Seynesiella juniperi*	I1186	MW405230	MW405222	N/A	MW619848	MW619846
*Spirosphaera beverwijkiana*	CBS 469.66	HQ696657	HQ696657	N/A	N/A	N/A
*Spirosphaera minuta*	CBS 476.66	HQ696659	HQ696659	N/A	N/A	N/A
*Stomiopeltis betulae*	CBS 114420	GU214701	GU214701	N/A	N/A	N/A
***Sympodiella multiseptata***	**CBS 566.71**	**MH872028**	**MH860264**	**N/A**	**N/A**	**N/A**
***Sympoventuria capensis***	**CBS 120136**	**KF156104**	**DQ885906**	**N/A**	**N/A**	**N/A**
*Trichodelitschia bisporula*	CBS 262.69	MH871039	MH859305	N/A	N/A	N/A
*Triscelophorus anisopteriodeus*	CGMCC 3.18978	MK577818	MK569511	MK577803	N/A	MK577833
*Triscelophorus konajensis*	YMF 1-04065	MK577820	MK569513	MK577805	MK607179	MK577835
***Tumidispora shoreae***	**MFLUCC 14-0574**	**KT314074**	**N/A**	**KT314075**	**N/A**	**N/A**
*Venturia inaequalis*	CBS 594.70	GU301879	KF156040	N/A	N/A	N/A
***Zeloasperisporium ficusicola***	**MFLUCC 15-0221**	**KT387733**	**N/A**	**KT387736**	**N/A**	**N/A**
***Zeloasperisporium hyphopodioides***	**CBS 218.95**	**EU035442**	**EU035442**	**N/A**	**N/A**	**N/A**
***Zeloasperisporium siamense***	**IFRDCC 2194**	**JQ036228**	**N/A**	**JQ036223**	**N/A**	**N/A**

Note: Ex-type strains are shown in bold, newly-generated sequences in bold and “N/A” indicates unavailable data in GenBank.

Phylogenetic analyses were conducted using RAxML-HPC v.8 on XSEDE (8.2.12), with a GTRGAMMA model and rapid bootstrap analysis followed by 1000 bootstrap replicates (Stamatakis 2014). Bayesian Inference (BI) analysis was performed by using MrBayes on XSEDE (3.2.7a) via CIPRES (Swofford 2002; Miller et al. 2010; Ronquist et al. 2012). The aligned FASTA file was converted to NEXUS format using AliView (Daniel et al. 2010). The best-fit evolutionary model for the individual dataset was determined using MrModelTest v. 2.3. 10 (Nylander 2004). The best-fit models for the BI analysis were GTR+I+G for LSU, SSU, *tef*1-α and *rpb*2 gene regions and SYM+I+G for the ITS gene region. The posterior probabilities (BI) were determined, based on Bayesian Markov Chain Monte Carlo (BMCMC) sampling (Huelsenbeck and Ronquist 2001). Two simultaneous Markov chains were run for 10,000,000 generations and trees were sampled every 1,000^th^ generation. The burn-in phase was set at 25% and the remaining trees were used for calculating posterior probabilities (BI).

Phylogenetic trees were visualised using FigTree version 1.4.4, and further edited using Adobe Photoshop 2019 programme (Adobe Systems, USA) and Adobe Illustrator version 51.1052.0.0 (Adobe Inc., San Jose, California, USA).

## Phylogenetic results

The phylogenetic positions of the four novel strains were inferred, based on combined LSU, ITS, SSU, *tef*1-α and *rpb*2 sequence data. The concatenated alignment comprised 4,307 characters (LSU: 1–859, ITS: 860–1,508, SSU: 1,509–2,515, *tef*1-α: 2,516–3,272, and *rpb*2: 3,273–4,307) from 62 taxa. Phylogenetic analyses were performed using Maximum Likelihood (ML) and Bayesian Inference (BI), with *Kirschsteiniothelia acutispora* (MFLU 21-0127) and *K. lignicola* (MFLUCC 10-0036) designated as outgroup taxa. Estimated base frequencies were A = 0.260487, C = 0.227430, G = 0.276721 and T = 0.235362, while substitution rates were AC = 1.128363, AG = 2.507625, AT = 1.588330, CG = 0.965261, CT = 4.664676 and GT = 1.000000. The gamma distribution shape parameter (α) was 0.359496.

**Figure 1.**
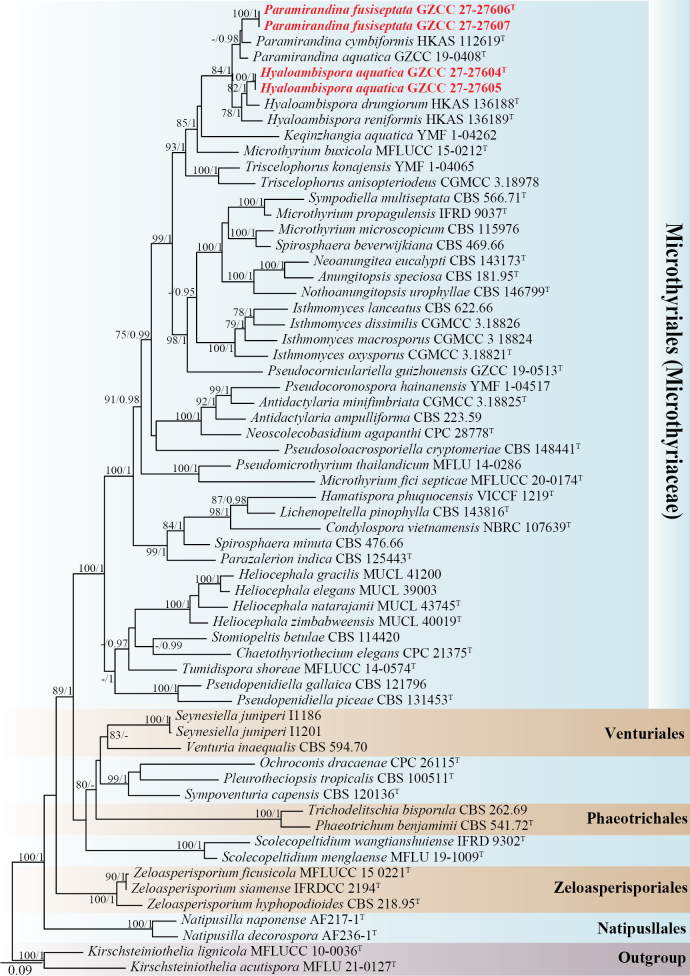
Phylogenetic tree generated from the RAxML analysis, based on the combined LSU, ITS, SSU, *tef*1-α and *rpb*2 sequence data. Bootstrap support values of RAxML (ML) equal to or greater than 75% and Bayesian posterior probabilities (BYPP) equal to or greater than 0.95 are given near the nodes as ML/BYPP, respectively. The Maximum Likelihood (ML) and Bayesian Inference (BI) analyses yielded similar tree topologies. A hyphen (“-”) denotes support values below 75% for ML and posterior probabilities below 0.95 for BI. *Kirschsteiniothelia acutispora* (MFLU 21-0127) and *K. lignicola* (MFLUCC 10-0036) were selected as outgroups. Ex-type strains are indicated by “^T^” and newly-obtained isolates are shown in bold red.

Based on the concatenated LSU, ITS, SSU, *tef*1-α and *rpb*2 phylogenetic analysis (Fig. 1), our collections belong to *Hyaloambispora* and *Paramirandina* within Microthyriaceae (Microthyriales). Isolates GZCC 27-27604 and GZCC 27-27605 formed a well-supported clade (82% ML/BYPP = 1), sister to *Hyaloambispora drungiorum* (HKAS 136188). Additionally, isolates GZCC 27-27606 and GZCC 27-27607 formed a sister clade to *Paramirandina cymbiformis* (HKAS 112619), with weak support.

### Taxonomy

#### Hyaloambispora aquatica

Taxon classificationFungiMicrothyrialesMicrothyriaceae

L. Shao & J. Ma sp. nov.

45CD4F17-2D66-598E-B894-174E457D8B4E

905781

Fig. 2

##### Etymology.

‘‘*aquatica*” refers to the occurrence of this species in an aquatic habitat.

**Figure 2.**
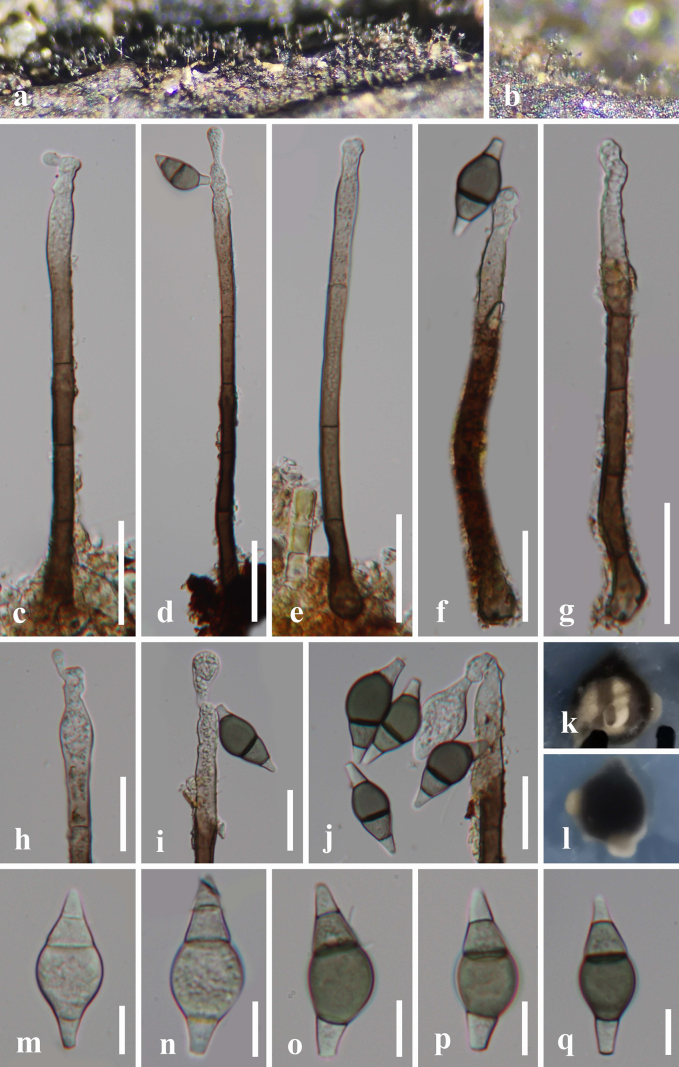
*Hyaloambispora aquatica* (GZAAS 25-0795, holotype). **a, b**. Colonies on the host surface; **c–g**. Conidiophores, conidiogenous cells and conidia; **h–j**. Conidiogenous cells and conidia; **k, l**. Colonies on PDA from above and below; **m–q**. Conidia. Scale bars: 30 μm (**c–g**); 20 μm (**h–j**); 10 μm (**m–q**).

##### Holotype.

GZAAS 25-0795.

##### Description.

***Saprobic*** on submerged decaying wood in freshwater river. ***Asexual morph*: *Colonies*** superficial, effuse, hairy or velvety, aggregated into fascicles, pale brown to brown. ***Mycelium*** immersed, consisting of hyaline to pale brown, smooth-walled, branched, septate, smooth hyphae. ***Conidiophores*** 119.5–167.5 × 7.5–10 μm (x̄ = 138 × 8.5 μm, n = 25), macronematous, mononematous, solitary, erect, unbranched, septate, straight or flexuous, cylindrical, slightly constricted at septa, dark brown at the base, subhyaline to pale brown towards the apex. ***Conidiogenous cells*** 43–68 × 6–8.5 μm (x̄ = 54 × 7 μm, n = 25), polyblastic, sympodial, terminal, becoming intercalary, percurrent, denticulate, subhyaline to pale brown, bearing tiny, protuberant, circular scars, sometimes with percurrent proliferation and slightly constricted at septa. ***Conidia*** 24–34 × 10.5–13.5 μm (x̄ = 30.5 × 12 μm, n = 35), acropleurogenous, fusiform, reniform or obpyriform, straight or slightly curved, smooth-walled, 3-euseptate, swollen in the central cell, central cell olivaceous or pale brown to brown, apical cell subhyaline, conical or slightly rounded, basal cell subhyaline, hyaline when young, truncate. ***Sexual morph***: Undetermined.

##### Culture characteristics.

Conidia germinating on PDA within 28 hours, producing germ tubes from the conidial body. Colony on PDA reaching 13 mm diam. after 45 days at room temperature (approximately 25 °C), circular or irregular, umbonate, with undulate margin; pale brown to dark brown, reverse side displays pale brown to dark brown colonies.

##### Material examined.

China, • Hainan Province, Dongfang City, on submerged decaying wood in a freshwater habitat, 22 May 2025, Jian Ma, DF13 (GZAAS 25-0795, holotype), ex-type living cultures GZCC 27-27604; *ibid*., DF18 (GZAAS 25-0796, paratype), living culture GZCC 27-27605.

##### Notes.

The specimen (GZAAS 25-0795) exhibits morphological similarities to *Hyaloambispora drungiorum* (HKAS 136188) in terms of conidiophore and conidial morphology, as described by Xu et al. (2025). However, GZAAS 25-0795 differs from *H. drungiorum* (HKAS 136188) in having shorter conidiophores (119.5–167.5 μm vs. 404–580 μm) (Xu et al. 2025). In addition, *H. drungiorum* possesses guttulate conidia, whereas this characteristic is absent in GZAAS 25-0795 (Xu et al. 2025). Moreover, phylogenetic analyses recovered our isolates (GZCC 27-27604 and GZCC 27-27605) as sister to *H. drungiorum* (HKAS 136188), with 82% ML/1 BYPP support. Additionally, base pair comparisons between *Hyaloambispora aquatica* (GZCC 27-27604, ex-type) and *H. drungiorum* (KUNCC 10416, ex-type) reveal 17/746 bp differences in LSU (2.3%, including one gap). Therefore, based on both LSU, ITS, SSU, *tef*1-α and *rpb*2 sequence data and morphological differences, we introduce *Hyaloambispora aquatica* as a novel species.

#### Paramirandina fusiseptata

Taxon classificationFungiMicrothyrialesMicrothyriaceae

L. Shao & J. Ma sp. nov.

F402036C-E575-5616-82A0-54E88B080292

905782

Fig. 3

##### Etymology.

‘‘*fusiseptata*” refers to the fusiform, septate conidia, characterised by a pale brown to brown central cell.

**Figure 3.**
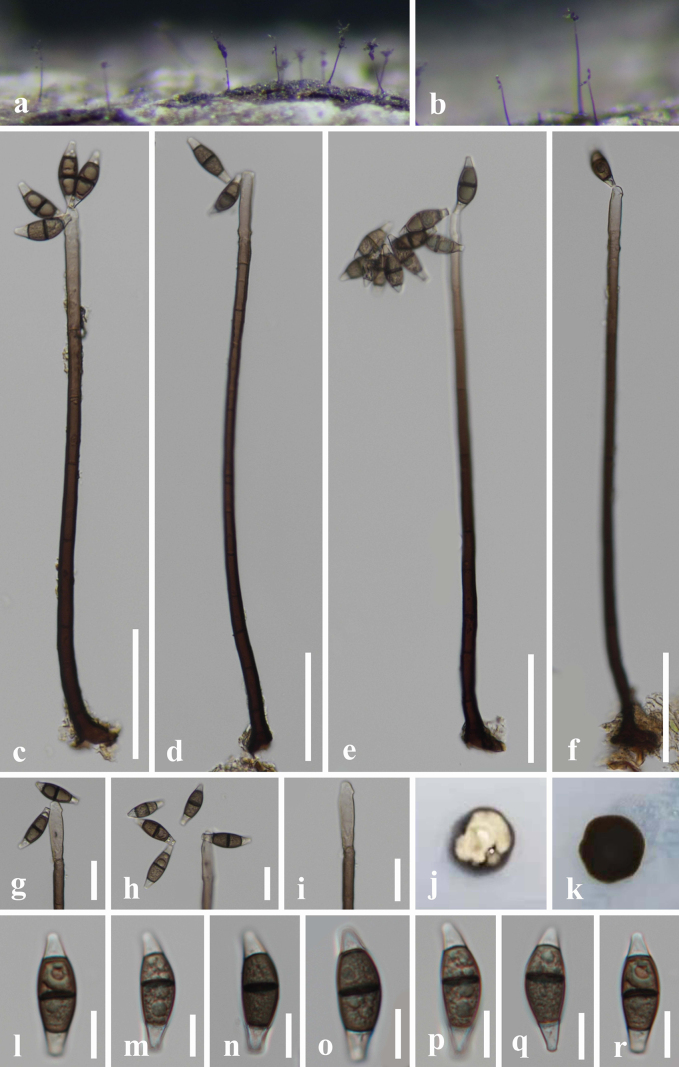
*Paramirandina fusiseptata* (GZAAS 25-0797, holotype). **a, b**. Colonies on the host surface; **c–f**. Conidiophores, conidiogenous cells and conidia; **g, h**. Conidiogenous cells and conidia; **i**. Conidiogenous cells; **j, k**. Colonies on PDA from above and below; **l–r**. Conidia. Scale bars: 50 μm (**c–f**); 20 μm (**g–i**); 10 μm (**l–r**).

##### Holotype.

GZAAS 25-0597.

##### Description.

***Saprobic*** on submerged decaying wood in freshwater river. ***Asexual morph*: *Colonies*** superficial, effuse, hairy or velvety, aggregated into fascicles, pale brown to brown. ***Mycelium*** immersed, consisting of hyaline to pale brown, smooth-walled, branched, septate, smooth hyphae. ***Conidiophores*** 208.5–273 × 6.5–10 μm (x̄ = 253.5 × 7.5 μm, n = 25), macronematous, mononematous, solitary, erect, unbranched, septate, straight or flexuous, cylindrical, slightly constricted at septa, dark brown at the base, subhyaline to pale brown towards the apex. ***Conidiogenous cells*** 35–53.5 × 4.5–7 μm (x̄ = 44.5 × 6 μm, n = 25), polyblastic, sympodial, terminal, becoming intercalary, percurrent, denticulate, subhyaline to pale brown, bearing tiny, protuberant, circular scars, sometimes with percurrent proliferation and slightly constricted at septa. ***Conidia*** 20.5–28 × 7.5–9 μm (x̄ = 25 × 8.5 μm, n = 30), acropleurogenous, fusiform, straight or slightly curved, smooth-walled, 3-euseptate, central cell pale brown to brown, apical cell subhyaline, conical or slightly rounded, basal cell subhyaline, truncate. ***Sexual morph***: Undetermined.

##### Culture characteristics.

Conidia germinating on PDA within 32 hours, producing germ tubes from the conidial body. Colony on PDA reaching 12 mm diam. after 42 days at room temperature (approximately 25 °C), circular, flat, with entire margin; subhyaline, pale brown to brown, reverse side displays yellowish-brown to dark brown colonies.

##### Material examined.

China, • Guizhou Province, Qiannan Buyi and Miao Autonomous Prefecture, Longli County, on decaying wood in a freshwater habitat, 2 September 2021, Jian Ma, SZ120 (GZAAS 25-0797, holotype), ex-type living cultures GZCC 27-27606; *ibid*., SZ22 (GZAAS 25-0798, paratype), living culture GZCC 27-27607.

##### Notes.

According to BLASTn searches of the NCBI GenBank database, the ITS, LSU, *tef*1-α and *rpb*2 sequences of the new isolates (GZCC 27-27606 and GZCC 27-27607) showed the highest similarities to *Paramirandina aquatica* (GZCC 19-0408), *Pleurotheciopsis bramleyi* (CBS 127863) and *P. cymbiformis* (HKAS 112619). The ITS sequence shared 90.6% similarity with *P. aquatica* (GZCC 19-0408), the LSU sequence shared 99.64% similarity with *Pleurotheciopsis bramleyi* (CBS 127863), while the *tef*1-α and *rpb*2 sequences shared 94.59% and 92.34% similarity, respectively, with *P. cymbiformis* (HKAS 112619). All comparisons were based on 100% query coverage. Our phylogenetic analysis supports that our isolates (GZCC 27-27606 and GZCC 27-27607) formed a sister clade to *Paramirandina cymbiformis* (HKAS 112619), with weak support. Morphologically, *P. fusiseptata* (GZAAS 25-0797) differs from *P. aquatica* (GZAAS 20-0303) and *P. cymbiformis* (HKAS 112619) in its conidial morphology, having 3-euseptate conidia with a pale brown to brown central cell and a subhyaline apical cell, whereas the conidia of the previously described species of *Paramirandina* are hyaline and 2–6-septate (Liu et al. 2023). Additionally, *P. fusiseptata* differs from *Pleurotheciopsis bramleyi* in its conidial morphology by possessing conidia with a pale brown to brown central cell and a subhyaline basal cell, whereas those of *P. bramleyi* are hyaline, catenate and produced from denticles (Sutton 1973). Therefore, despite the marked morphological differences, phylogenetic analyses, based on ITS, LSU, SSU, *tef*1-α and *rpb*2 sequence data, combined with morphological characteristics, indicate that strains GZCC 27-27606 and GZCC 27-27607 belong to *Paramirandina*. We therefore tentatively assign these strains to this genus and introduce them as a new species, *Paramirandina fusiseptata*.

## Discussion

The two species we introduce, *Hyaloambispora aquatica* and *Paramirandina fusiseptata*, are morphologically highly similar to genera *Nakatopsis* and *Pleurotheciopsis* (Sutton 1973; Cazau et al. 1993; Castañeda Ruíz and Iturriaga 1999; Castañeda Ruíz et al. 2001; Whitton et al. 2001; Ai et al. 2019). For example, *Paramirandina fusiseptata* shares features with *Pleurotheciopsis sylvestris*, *P. triseptata* and *P. websteri*, including macronematous, mononematous conidiophores; polyblastic, monoblastic, sympodial, intercalary conidiogenous cells; and 3-septate, fusiform, straight to slightly curved conidia (Sutton 1973; Cazau et al. 1993; Castañeda Ruíz and Iturriaga 1999; Castañeda Ruíz et al. 2001; Ai et al. 2019; Xu et al. 2025). Similarly, *Hyaloambispora aquatica* resembles *Nakatopsis malaysiana* in possessing macronematous, mononematous conidiophores; polyblastic, monoblastic, sympodial, intercalary conidiogenous cells; and acropleurogenous, fusiform, reniform or obpyriform, straight to slightly curved, smooth-walled conidia that are swollen in the central cell (Whitton et al. 2001; Xu et al. 2025).

The genera *Hyaloambispora*, *Paramirandina* and *Pleurotheciopsis* share similar macronematous, mononematous conidiophores with integrated conidiogenous cells; however, they can be distinguished by the morphology of their type species. *Hyaloambispora* is characterised by 3-septate, smooth-walled conidia that are fusiform, reniform, obpyriform or cymbiform, with a swollen central cell, a pale brown to brown central region and a subhyaline apical cell. In contrast, the type species of *Paramirandina* produces hyaline, 2–6-septate conidia that are solitary or catenate, fusiform, cymbiform or narrowly lunate, representing a distinctly different conidial morphology. The type species of *Pleurotheciopsis* is characterised by hyaline, catenate, 0–3-septate conidia developing from the tips of denticles. Molecular phylogenetic analyses further support the separation of *Hyaloambispora* and *Paramirandina*, although the phylogenetic position of *Paramirandina* remains insufficiently resolved due to limited multilocus sequence data. Similarly, the taxonomic status of *Pleurotheciopsis* remains uncertain because molecular data are available for only a few taxa and the phylogenetic placement of its type species has not been robustly resolved (Sutton 1973; Cazau et al. 1993; Castañeda Ruíz and Iturriaga 1999; Castañeda Ruíz et al. 2001; Ai et al. 2019; Xu et al. 2025). Therefore, although morphological features of the type species support the recognition of *Paramirandina* and *Pleurotheciopsis* as distinct genera, additional collections of authentic material and comprehensive multigene phylogenetic analyses are required to clarify their generic circumscriptions and evolutionary relationships.

*Pleurotheciopsis* was established by Sutton (1973), with *P. pusilla* designated as the type species. Currently, seven species are accepted in the genus (Sutton 1973; Cazau et al. 1993; Castañeda Ruíz and Iturriaga 1999; Castañeda Ruíz et al. 2001; Ai et al. 2019). Vu et al. (2019) deposited molecular sequence data for *Pleurotheciopsis bramleyi* and *P. tropicalis* in GenBank; however, no accompanying phylogenetic analyses or detailed morphological assessments were provided. In our phylogenetic analyses (Fig. 1), *P. tropicalis* clustered with *Ochroconis dracaenae* rather than with species of *Hyaloambispora* or *Paramirandina*. Furthermore, the authenticity of the sequences attributed to *P. bramleyi* and *P. tropicalis* should be verified through examination of the corresponding voucher specimens, collection metadata and evidence of deposition in publicly accessible culture collections.

## Supplementary Material

XML Treatment for Hyaloambispora aquatica

XML Treatment for Paramirandina fusiseptata

## Additional information

### Artificial Intelligence (AI) use

The authors accept full responsibility for the content of the manuscript, including the disclosure of any use of AI.

No AI tools were used in the preparation of this manuscript.

### Author ORCIDs

L. Shao https://orcid.org/0000-0002-2196-1850

Y. Liu https://orcid.org/0009-0003-8807-533X

Y. Shi https://orcid.org/0009-0001-1976-8883

Y. Liu https://orcid.org/0009-0008-3000-5394

W. Yang https://orcid.org/0009-0007-7773-1337

J. Ma https://orcid.org/0009-0008-1291-640X

### Data availability

All of the data that support the findings of this study are available in the main text.
